# Re-engineering The Clinical Research Enterprise in Response to COVID-19: The Clinical Translational Science Award (CTSA) experience and proposed playbook for future pandemics

**DOI:** 10.1017/cts.2021.10

**Published:** 2021-02-18

**Authors:** Barry S. Coller, John B. Buse, Robert P. Kimberly, William G. Powderly, Martin S. Zand

**Affiliations:** 1 Allen and Frances Adler Laboratory of Vascular Biology, Rockefeller University, New York, NY, USA; 2 Department of Medicine, University of North Carolina School of Medicine, Chapel Hill, NC, USA; 3 Department of Medicine, University of Alabama at Birmingham, Birmingham, AL, USA; 4 Division of Infectious Diseases and Institute for Public Health, Washington University in St. Louis, St. Louis, MO, USA; 5 Department of Medicine, University of Rochester Medical Center, Department of Medicine, Nephrology, Rochester, NY, USA

**Keywords:** COVID-19, CTSA, clinical research, pandemic, translational research

## Abstract

The 2020 COVID-19 pandemic has had a profound impact on the clinical research enterprises at the 60 Clinical and Translational Science Award (CTSA) Hubs throughout the nation. There was simultaneously a need to expand research to obtain crucial data about disease prognosis and therapy and enormous limitations on conducting research as localities and institutions limited travel and person-to-person contact. These imperatives resulted in major changes in the way research was conducted, including expediting Institutional Review Board review, shifting to remote interactions with participants, centralizing decision-making in prioritizing research protocols, establishing biobanks, adopting novel informatics platforms, and distributing study drugs in unconventional ways. National CTSA Steering Committee meetings provided an opportunity to share best practices and develop the idea of capturing the CTSA program experiences in a series of papers. Here we bring together the recommendations from those papers in a list of specific actions that research sites can take to strengthen operations and prepare for similar future public health emergencies. Most importantly, creative innovations developed in response to the COVID-19 pandemic deserve serious consideration for adoption as new standards, thus converting the painful trauma of the pandemic into “post-traumatic growth” that makes the clinical research enterprise stronger, more resilient, and more effective.

## Introduction

The first four cases of what would later be called severe acute respiratory syndrome-related coronavirus 2 (SARS-CoV-2) infection, or COVID-19, were reported in Wuhan, China, on December 31, 2019 [1,2], although later studies indicate that individuals probably were infected earlier, perhaps even in November [3–5]. The nucleotide sequence of the virus was made public by a consortium of Chinese and Australian institutions on January 10, 2020 [6], establishing the causative agent as a coronavirus with genetic similarities to SARS-CoV and Middle East Respiratory Syndrome (MERS). Although its mode of transmission was unclear, early anecdotal reports indicated that single patients could infect large numbers of health care workers, raising the likelihood that person-to-person spread could occur readily, which was confirmed by January 21, 2020 [7]. Later, it was appreciated that asymptomatic individuals could spread the virus to large numbers of contacts outside of the health care setting [8]. The first US case was reported in a traveler who returned to Washington State from Wuhan on January 15, 2020; the first European cases of COVID-19 were reported on January 24 in France [9]. The US partially restricted travel to the United States from China on January 30 and declared the disease a Public Health Emergency on January 31. On the same day federal officials ordered a 14-day quarantine for a group of 195 citizens repatriated from China, but did not restrict travel from Europe.

Based on the DNA sequence of the virus a polymeric chain reaction test to detect SARS-CoV-2 viral DNA was developed by German investigators at Berlin’s Charité Hospital [10] and later adopted by the WHO, but not by the US CDC or Food and Drug Administration (FDA). The CDC prepared its own test but encountered substantial delays, thus seriously limiting and delaying testing in the United States [11,12]. For example, the CDC website on March 1 indicated it could perform only 350 tests per day and that it had material for only 75,000 tests for the entire country. In contrast, by March 16, the WHO had already distributed 1.5 million tests to 120 countries [13]. Data from Italy, Japan, South Korea, and Iran in early March demonstrated the enormously rapid spread of the virus, which overwhelmed medical facilities and taxed health care professionals to their limits. The WHO declared COVID-19 a pandemic on March 11 and that was soon followed by outbreaks in New York, New Jersey, Connecticut, Massachusetts, and other states. For example, on March 2, the US reported just 16 new cases that day, but by March 30, it reported 21,469 new cases.

## The Clinical and Translational Science Award (CTSA) Consortium’s Response

**Fig. 1.**
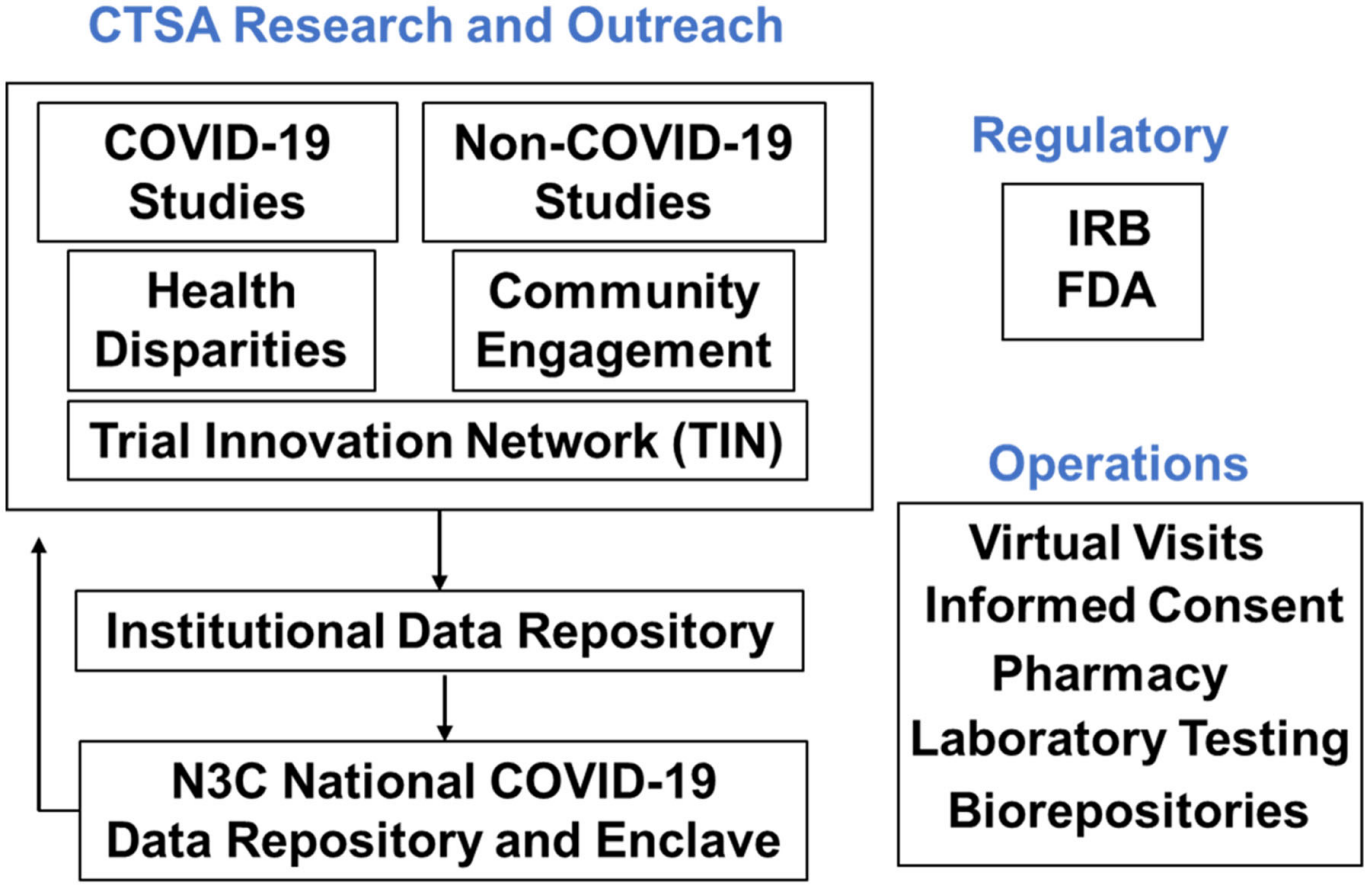
**CTSAs and COVID-19.** Major elements of the translational research enterprise at Clinical and Translational Award (CTSA) hubs and nationally related to the COVID-19 pandemic. FDA, US Food and Drug Administration; IRB, Institutional Review Board; N3C, National COVID Cohort Collaborative.

The National Institutes of Health (NIH) through its National Center for Advancing Translational Science (NCATS) supports approximately 60 academic hubs in its CTSA program. Nearly all of the CTSA hubs are affiliated with one or more health care systems across the country and are committed to the goal of enhancing national capacity, methods, and processes in clinical and translational research, focusing on the local needs of their communities. As the COVID-19 pandemic spread, the national CTSA Steering Committee devoted increasing attention to sharing best practices among CTSA sites as each hub addressed the challenges it faced in responding to the local manifestations of the pandemic, and as the Steering Committee collectively focused on advancing discovery and translational science targeting COVID-19 broadly. From those Steering Committee meetings emerged plans to capture the creative ideas developed by CTSA hubs in a series of papers focusing on different elements in the clinical research enterprise (Fig. 1). The outcome is this special issue of the Journal of Clinical and Translational Sciences.

Each paper is flexibly organized by the following template:

1. What were the practices before the COVID-19 pandemic?
2. How were practices altered/redefined/modified/streamlined to address the challenges and exigencies of the COVID-19 pandemic?
3. What were the key lessons learned?
4. Which, if any, extraordinary practices developed as a response to COVID-19 should now become standard, and which, if any raised sufficient concerns that they should not be continued or perhaps even considered in the future in the face of a similar public health challenge?
5. If a similar public health challenge occurred in the future, what would be the sequence of actions you recommend to take in response?

We coupled this initiative with an extensive survey of the CTSA hubs created by the University of Rochester Center for Leading Innovation and Collaboration (CLIC) Survey Team by integrating the questions proposed by the each of the writing groups (Supplementary Table). A total of 60 hubs responded, providing robust data about measures that the hubs implemented, along with an assessment of best practices. The detailed results of the survey are included in the individual manuscripts. The most dramatic finding was that 87% of the CTSA hubs indicated that they were involved in creating institutional COVID-19-related governing policies, highlighting the importance of CTSAs in shaping the clinical research enterprise.

This theme issue of *The Journal of Clinical Translational Science* brings all of these papers together, and this paper provides a summary of the key recommendations proposed by the authors of each of the papers. We have focused our recommendations on things to do now (Table) based on the CTSA experience and best practices because careful planning now will facilitate and speed implementation of measures to address a future public health challenge. The same table should serve as a checklist if we are confronted with another public health emergency of comparable magnitude. In this way, we hope to provide a “playbook” as a resource for those called upon to lead the clinical research enterprise at their institution in response to new public local, regional, or global health crises.

The topics covered in Table 1 are wide ranging because major public health emergencies impact virtually every phase of research operations. The impact of COVID-19 on educational programs has been particularly profound, requiring an enormous effort to sustain trainees’ productivity. This topic is being addressed in a separate publication by the CTSA career and workforce development group [14]. Based on a survey of TL1 trainees and KL2 scholars, they found that lack of access to research facilities, clinics, team members, and human subjects, coupled with the need for homeschooling, were major challenges. Strategies to maintain research productivity involved trying to focus on writing, time management, virtual connections with colleagues, and shifting to research activities not requiring laboratory/clinic settings. They also described serious concerns by trainees and scholars that their decreased productivity during the pandemic might have a negative impact on their long-term research and career goals and their ability to obtain research funding. They offered a series of actions that programs, institutions, and the NIH can take to mitigate the impact of the pandemic on translational trainees and scholars.

**Table 1.** Things to do now and a Checklist for a future similar public health emergency

I. Institutional Administration A. Create policies to align health system, hospital, university, and medical school senior administrations, along with legal departments, Institutional Review Boards (IRBs), and technology transfer departments, to expedite decision-making related to clinical and research operations in the event of a major sustained public health emergency. B. Create Advisory Boards and Develop Charters and Policies 1. Clinical Research Prioritization Advisory Board. Establish criteria for prioritization of research studies, including those related to the pandemic and those unrelated to the pandemic. For studies related to the pandemic, these may include, for example, institutional priorities; resource Intensity; patient availability; staff capacity; minimization of duplication of studies; clinical equipoise; consent; statistical power to be informative; and adequate funding. For studies unrelated to the pandemic, criteria may include whether it involves a life-threatening disease, whether participants already enrolled need continuing access to otherwise unavailable drugs or other treatments, whether the studies are time-limited by virtue of a sponsor’s requirements, and whether the studies are crucial for the career development of trainees who have time-limited appointments. 2. Biorepository Scientific Advisory Board. 1) Establish principles related to organization (centralized, federated, and/or decentralized); potential modifications for consent (patient, legal representative if incapacitated, next of kin for autopsy); availability of translated consent documents; potential proactive sample collection priorities even if there is no current protocol (e.g., health care workers, pediatric samples, convalescent patients); potential specimen types and volume; processing standards; facilities; linkage to clinical data and informatics support; tracking and storage; retrieval; and distribution. 2) Communicate with and engage all stakeholders to minimize individual investigator resistance in the future. 3) Develop a broad consent document to encompass all potential uses of samples. 3. Biorepository Governance Advisory Board. Establish principles of operations: biosafety, including availability and access to BSL2+ and BSL3 facilities; personnel training; regulatory and legal aspects; data sharing; sample sharing (academic investigators, government investigators, industry). 4. Institutional Safety Board. Assess on regular basis the latest information on safety risks to patients, health care workers, supporting staff, students, and others, and develop and modify policies and procedures to maximize protections accordingly. Charge the Board with making quantitative recommendations at timely intervals for stockpiling of personal protective equipment (PPE) and identifying restocking supply lines, including expectations for the need for redundancy in suppliers. If institution needs to shut down, use Institutional Safety Board as core for a Reopening Committee. 5. Research Support Board. Create a Pilot Project Program and review proposals and disperse funds rapidly. Integrate efforts with Technology Transfer Department to ensure that all intellectual property protection and licensing opportunities are maximized. II. Individual Departments and Units A. Communications Department 1. Develop plan for communicating with staff, faculty, students, house staff, and institutional supporters through a variety of different media, including town hall meeting (in person or virtual), email, messaging, and social media, 2. Design websites related to clinical activities, research activities, community engagement information, and others. 3. Plan for communication with public-facing media, including radio, television, and social media about the essential role well-designed clinical studies play in selecting the safest and most effective preventive and therapeutic interventions. B. Community Engagement and Health Disparities Programs 1. Strengthen bidirectional partnerships with community organizations and community leaders. 2. Strengthen and support community health workers by providing resources, training, access to technology, and ability to gain access to medical care for patients in referral centers. 3. Develop strategy, policies, and procedures for measuring relative impact of public health emergencies in neighboring populations at high risk by virtue of poverty, population density, racism, occupations (especially health care workers and other essential personnel), environmental air pollution, and other relevant factors. 4. Develop language-appropriate translations of all important communications. 5. Ensure that electronic health records (EHR) include sufficiently granular race and ethnicity data to assess disproportionate impact of disease on specific groups. 6. Develop a proactive plan with community leaders to provide a steady stream of accurate medical information and dispel misinformation in the community. 7. Develop a proactive plan with community, government, and industry partners to diminish the “digital divide” by supporting programs to make internet access and computers broadly available to individuals for health care information. 8. Maximize the communication value of patient portals for transmitting health information. 9. Plan with local public health agencies to bring needed services directly into the community, including diagnostic testing and long-term follow-up. C. Development Department. Prepare a draft of a campaign for philanthropic support of basic and clinical research for rapid implementation. D. Human Resources Department. 1. Develop a plan to address need for increased support of essential personnel with pre-school and school-age children. 2. Develop a plan for potential redeployment of personnel and craft cross-training experiences to expedite redeployment if necessary. 3. Review policies for sick leave if need to quarantine. 4. Consider developing “Work from Anywhere” policies for rapidly hiring individuals with required skills, such as informatics. E. Information Technology (IT) Department 1. Strengthen informatics platforms to ensure security, privacy, and technical capability to support rapid expansion of virtual operations, including tele-medicine, institutional operations, educational activities, and both basic and clinical research, including eConsent and documentation of eConsent. 2. Develop customized dashboards for different Clinical and Translational Award (CTSA) hub leaders and institutional leaders based on data required to manage pandemic preparedness and pandemic operations. 3. Participate in the N3C data enclave that links the EHRs on individuals diagnosed with COVID-19 from multiple sites so that investigators can search the records to answer key questions about the diagnosis, prognosis, and treatment of the disorder. 4. Partner with state and local public health departments, health exchanges, claims databases (CMS Medicare and Medicaid) to integrate informatics platforms to address public health issues such as vaccination records. Assess needs for data reporting to regional and national agencies. 5. Work with clinical teams and institutional leadership to align as best as possible the information clinicians require for patient management and research (clinical decision support with predictive tools, cohort identification, grant submissions, response to new experimental therapies) and institutional leaders require for global planning (intensive care unit [ICU] beds, ventilators, PPE) versus the data collected by current systems. Adjust as necessary to better align the needed and collected data. 6. Work with legal department to speed implementation of data use agreements and memoranda of understanding. 7. Review cybersecurity protections in the face of urgent requests for data sharing. F. Legal Department 1. Prepare to expedite contracting for clinical trials with industry by exploring master agreements that can be rapidly implemented. 2. Review legal aspects related to security and HIPAA with rapid expansion of virtual operations. G. Research Pharmacy 1. Stockpile pharmacy-specific PPE and identify potential alternative suppliers if encounter a shortage. 2. Develop policies for emergent, expedited processes for developing drug profiles and computerized physician order entry screens for novel experimental drugs and repurposed drugs. Ensure availability to dispense drugs 24/7/365 for studies requiring rapid drug initiation. 3. Assess current capacity for formulating novel experimental agents and assess potential value and cost of expanding capabilities. 4. Assess relationships with network pharmacists and whether to expand relationships in anticipation of future needs for drug distribution in an emergency. 5. Develop policies for delivering experimental drugs to participants in research studies under emergency conditions, e.g., home delivery by hand or mail/messenger or curb-side or valet pick-up from site or collaborating network pharmacy. 6. Assess most likely medications to be in short supply as a result of a major public health emergency (e.g., medications used in ICUs) and consider stockpiling, identification of alternative suppliers, and creation of teams to modify and restrict utilization of drugs in short supply. For novel potentially life-saving medications in short supply, develop policies with clinicians and bioethicists on criteria for fairest method to decide on distribution (e.g., drug lottery). 7. Work with community engagement groups on combating misinformation related to evidence of drug safety and efficacy. H. Sponsored Projects Department. 1. Develop a plan to systematically scan on a daily basis funding opportunities from government (federal, state, local), foundations, individual philanthropy, and industry. 2. Create a communication plan to disseminate funding information. 3. Plan for expansion of a cadre of sponsored research personnel to facilitate timely grant proposal submissions. I. Technology Transfer Department 1. Prepare to expedite Material Transfer Agreements (MTAs) for investigators at other institutions who may want access to Biorepository samples and for institutional investigators who may want access to samples at other institutions by developing master MTAs with institutions most likely to want or provide such samples. 2. Monitor Pilot Research Projects related to the public health emergency for potential intellectual property and licensing opportunities. III. Human Research Protection Plan and IRB A. Review policies and procedures, and if necessary, add sections describing actions to be taken in response to a public health emergency: a. Criteria for differentiating Public Health Surveillance projects from Human Subjects Research; b. Temporary expansion of IRB capacity by adding personnel, reassigning and prioritizing reviews among existing IRBs, and/or creating a new IRB to address protocols related to the emergency; c. Developing and updating a roster of past IRB members and IRB staff who may be willing to volunteer to return to active duty to help address the need for rapid review of protocols; d. Triage of protocols based on their need for rapid review; e. Analysis of impact of single IRB review requirements on review mechanism, with potential to petition for exemption when supported by specific circumstances; f. Potential modification of general principle of limiting participation to a single experimental protocol. g. Impact of family members not being allowed to visit patients in hospital on informed consent process. h. Proactive assistance to inexperienced investigators in developing protocols requiring IRB review. i. Importance of insuring equitable and appropriate recruitment of research participants from groups disproportionately affected by the emergency through availability of translation of consent documents and community outreach measures. j. Ethical considerations guiding initiation and continuation of randomized clinical trials in the face of rapidly emerging clinical information. B. Integrate IRB efforts to speed review with efforts by Clinical Research Prioritization Advisory Board and others charged with expediting contracting, insurance coverage, budget negotiations, IND submissions, clinicaltrials.gov registration, obtaining and dispensing medications, and the creation of order sets in the EHR. Monitor the contributions of all of these activities to expediting the time from protocol development to first recruited participant. Consider instituting a Protocol Implementation Checklist to encourage an integrated approach and track relative contributions of each element to the process. C. Review policies and procedures related to informed consent: a. Acceptable platforms for communicating with participants and legal authorized representatives. b. Acceptable methods beyond signatures on paper informed consent forms for documenting agreement to participate in research. c. Accommodations when infection control does not allow collection of signed paper informed consent forms. d. The relative roles of translations of informed consent documents versus use of interpreters when there are time pressures to start studies and insure representative recruitment into studies. e. Studies involving off-label drugs vs single patient protocols. f. Need for (re-)consenting participants as they regain capacity when the initial consent was provided by a legally authorized representative. g. Provision of information related to consent by means other than in-person discussion between investigator or designee and research participant, such as videos. h. Review of research participants’ experiences with the unusual consent processes during the pandemic. i. Creation of patient and/or community advisory body to review benefits, disadvantages, and burdens of alternative consent processes employed during the pandemic. j. Assessment of the impact of different informed consent processes on equitable and appropriate recruitment, focusing on the potential contribution of the digital divide and cultural variations in trust in different processes. IV. Clinical and Translational Science Award (CTSA) Hub (Institutional Translational Center or Institute) 1. **Virtual operations.** Assess and strengthen ability to support virtual research operations: participant recruitment, informed consent, study management, study monitoring, physiologic measures, wearable sensors, laboratory sample collection, study visits, investigational product management, delivery of interventions, participant remuneration, site qualification, case report forms, and site initiation. Review potential electronic platforms with IT and research participants for HIPAA and 21 CFR Part 11 compliance, encryption, cybersecurity, privacy, and patient trust. Develop a range of options for study drug delivery: by hand to participant’s home, curbside or valet pick-up, shipping, home-health provider for parenteral medications, and medical delivery services. Create a Virtual Clinical Research website with resources and policies, including up to date US Food and Drug Administration (FDA) and US Department of Health and Human Services (HHS) guidances. 2. **Informed consent comprehension.** Assess, and if need be, build capacity to insure the integrity of the informed consent process through methods other than in-person meetings, e.g., videos, websites, podcasts, teleconference, telephone, multimedia, secure email, and secure text messaging. Engage research participants in developing strategies and tools. Provide training on new methods to research community. Assess the impact of the digital divide on the consent process. Plan for translation services to support each of the selected choices. 3. **Informed consent documentation.** Assess, and if need be, build capacity for capture of and documentation of consent through methods other than signatures on paper consent forms, e.g., waiver of signature, legal digital signature, picture of signed document, FDA MyStudies application, biological recognition, and electronic capture of oral consent. Engage research participants in developing strategies and tools. Provide training on new methods to research community. Assess the impact of the digital divide on the documentation of consent. Plan for translation services to support each of the selected choices. 4. **Interactions with the FDA.** Ensure capacity to submit documents to FDA electronically, preferably via electronic common technical document format. Develop ongoing training program for faculty and medical staff on FDA regulatory requirements and programs, including IND/IDE and Expanded Access programs (Emergency Use Authorization, Single Patient Protocols. Intermediate-Sized Patient Population Protocols, and Treatment Protocols). Develop policies to respond to a public health emergency by creating mechanisms for integrating efforts of regulatory support personnel, legal representatives, IRB leaders, and clinicians in prioritizing studies to receive regulatory support; frequent monitoring of changing FDA requirements and programs during the emergency; and reorganizing workflow to maximize support. Consider mechanisms to temporarily expand the number of experienced regulatory support personnel to meet the anticipated dramatic expansion of required services. 5. **Support of Rapid Publication of Results.** Provide assistance to investigators in preparing reports for publication and encourage investigators to accept invitations from journals to review manuscripts as rapidly as possible.

BSL, biosafety level; CMS, Centers for Medicare & Medicaid Services; CFR, Code of Feral Regulations; IND, Investigational New Drug; IDE, Investigational Device Exemption.

Many of the recommendations detailed in Table 1 are ones that are valuable to implement even without the threat of another public health emergency, highlighting that emergencies offer opportunities to garner broad support to implement things that would be beneficial under any conditions, but have faced one or more obstacles or have not risen to a high enough priority because of inertia. Some recommendations will require additional resources, which are likely to be in short supply as institutions face severe budgetary challenges, especially state medical schools that rely on public funds. This can potentially be balanced by an appeal for philanthropic support since the public now appreciates more than ever the vital role that translational research plays in protecting the health of the country and in developing novel diagnostic tests and therapies. The COVID-19 pandemic has provided the public with a thorough education in the roles of the NIH, FDA, and CDC, with otherwise esoteric topics such as Emergency Use Authorizations, and the statistical power of different clinical trial designs, now in the news on a regular basis. It has also more indirectly highlighted the crucial role of biomedical research, especially translational research, with vaccine development center stage in a way that has not occurred since the polio vaccine in the early 1950s [15]. This provides opportunities to build on the public interest with sustained public educational programs about the role and importance of translational research more broadly. This will also provide an opportunity to highlight the crucial role of the CTSA program in responding to the pandemic and other important health needs.

The CTSA program houses a Trial Innovation Network (TIN) to coordinate multisite clinical studies. The TIN has championed a number of innovative projects to support multiple COVID-19 studies transitioning to remote operations and the Recruitment Innovation Center (RIC) assisted investigators in engaging diverse communities via virtual Community Engaged Studios. The TIN also has played a role in maximizing the amount of information that can be extracted from studies of hydroxychloroquine and convalescent plasma and is playing an important role in implementing several NIH-supported studies for COVID-19. Similarly, the CTSA program is actively supporting the National COVID-19 Cohort Collaborative (N3C), a major initiative to link the electronic health records (EHRs) from patients with COVID-19 into a single searchable data enclave to speed the identification of important diagnostic, prognostic, and treatment information. The details of these initiatives are beyond the scope of this manuscript, but they are vital elements in a comprehensive response.

Perhaps the single greatest failing of the US response to the pandemic has been the fragmentation of clinical trials into mostly institution-specific units that have not been informative because of failure to meet the required number of participants. There have been complex challenges in sizing and completing trials, including tremendous variability in caseloads over short periods of time at individual sites, so that by the time protocols became approved the number of infections in the community dropped below the number expected and required for timely recruitment. On the flip side, when caseloads are exploding, and institutions require all hands on deck, with redeployment to active patient care roles of every person who can contribute to this mission, assigning personnel to support clinical trial recruitment and conduct is likely to be viewed as a “luxury” that the institution cannot afford to support. There is also the tension between making novel therapies generally available through FDA Expanded Access programs based on strong theoretical grounds, such as the use of convalescent plasma, and wanting to conduct rigorous randomized controlled clinical trials to unequivocally assess the value of the therapy. These challenges are not new, being clearly delineated by Upton Sinclair in his 1925 novel *Arrowsmith*, which was informed by the 1918 influenza pandemic [16].

These challenges cry out for immediate design and implementation of nation-wide multisite trials to answer as many questions as possible to optimize medical management as rapidly as possible. As we are writing, NIH is undertaking such an effort in its ACTIV [17] and CONNECTS [18] programs, with the CTSA TIN participating in the ACTIV-1 trial [19]. A full review of the effectiveness of these programs relative to actions taken in other countries, in particular the U.K. RECOVERY trial [20] and the ACCORD program [21], will provide valuable data for future planning. There is a need for national prioritization of the key scientific issues, and mechanisms to ensure adequate enrollment into the major studies by avoiding having multiple trials with overlapping enrolment criteria at many sites, and to prevent competition between NIH trials for sites and resources. Such an effort needs to extend beyond academic health centers to practice-based research networks if the largest number of people are to be enrolled in the shortest period of time and if the data are likely to be most generalizable. Now is the time to consider putting in place all of the necessary infrastructure, including the regulatory and legal documents and master protocols, as well as the organizational structure, that will allow for a true “turn-key” operation that can start enrollment within weeks of a major new public health emergency.

As an example, the lessons learned from the convalescent plasma therapy experience in the early phase of the COVID-19 pandemic indicate that the CTSA program as a consortium is ideally suited to rapidly develop a convalescent plasma program for future infectious disease challenges. By pooling resources and knowledge, it could rapidly in parallel: 1. Develop a high-throughput assay to measure antibody titers in convalescent plasma that correlate with viral neutralization. 2. Create a collection of validated biobank samples that could be used to analyze the sensitivity and specificity of the antibody assays. 3. Set up convalescent donor plasma collection centers in hub blood banks, regional blood collection facilities, and potentially in partnership with commercial plasma collection companies to obtain convalescent plasma anywhere in the country where the first convalescent patients reside, with distribution to sites around the country where it is needed most. Since it is crucial to obtain data from randomized studies to assess the potential benefits and risks of convalescent plasma, the CTSAs in collaboration with the TIN could create a master protocol, coupled with model subcontracts and a regulatory pathway, for rapid implementation in a future pandemic. Based on the currently available data from the COVID-19 experience, such a trial might best be limited to early institution of therapy with plasma containing high titers of antibody in an attempt to maximize the anticipated treatment effect. Such an effort would, however, also require funding, a secure supply chain, and the availability of staff who are not directly engaged in providing medical care to the patients. If such a mechanism was in place for the current pandemic, it is possible that high-quality data would have been available to assess the likely benefits and risks of convalescent plasma therapy within less than 5–6 months of the rapid expansion of the disease into the community. Thus, by combining the national reach of the CTSA program and its remarkable depth of full spectrum translational expertise, the consortium’s unique synergy could be put to maximal use in improving the health of the nation and the world.

## Discussion

The COVID-19 pandemic has rapidly emerged as one of the greatest translational research challenges in the last 100 years. It has changed nearly everything, from our ability to conduct in-person research visits to an unprecedented race for effective therapies and vaccines. In those areas of the country most severely affected by the virus, it has also required bravery, dedication, and courage on the part of countless medical professionals and other first responders as they put their own health at risk to save others, as well as the bravery of the research participants themselves. Despite the terrible toll that the pandemic has taken on both institutions and individuals, we believe that CTSA hubs have shown enormous creativity and perseverance to solve a myriad of new translational challenges, as well as team science on a scale that we could only imagine just a year ago. This response fits well the framework of Pandemic-Driven Post-traumatic Growth for Organizations and Individuals proposed by Olson et al. [22], defined as “positive psychological change experienced as a result of a struggle with highly challenging life circumstances,” providing an opportunity to view the pandemic not only as an unimaginable destructive force but also as a potential force for improvement. Among the key elements of the framework are, at the personal level, development of deeper relationships, openness to new possibilities, greater sense of personal strength, and greater appreciation of life. For success, it requires deliberative reflection, leading to action characterized by awareness, transparency, motivation, creativity, and dedication to improvement. Institutional parallels include transforming communication and delegation of authority and actively supporting caregivers.

Crisis management is often focused on restoring organizational function to its pre-crisis level, but perhaps a more appropriate goal is to achieve a higher level of function as a result of learning from the traumatic event. Reflective assessment, identification of extraordinary role models, identifying opportunities for reinvention of processes, contemplating how the experience connects the institution to the broader community and humanity, and reappraising priorities with regard to what is truly most important are some of the ways to achieve the goals of post-traumatic growth. There is no way to ignore the trauma we have all experienced individually and as members of great institutions. We hope that this theme issue detailing the activities of the CTSA program in response to the COVID-19 pandemic will help contribute to the goal before all of us, to make the trauma just the first chapter in the story of post-traumatic growth that will make all of our institutions and the CTSA program stronger, more resilient, and more effective.

## Supplementary material

For supplementary material accompanying this paper visit http://dx.doi.org/10.1017/cts.2021.10. click here to view supplementary material
