# Supplementary material for: Re-engineering The Clinical Research Enterprise in Response to COVID-19: The Clinical Translational Science Award (CTSA) experience and proposed playbook for future pandemics
Source: J Clin Transl Sci. 2021 Feb 18;5(1):e96. doi: 10.1017/cts.2021.10 (PMC8137226; doi:10.1017/cts.2021.10)
Supplement: Supplementary file 1 [file S2059866121000108sup001.pdf]

CTSA Clinical & Translational Science Awards Program

CLIC Center for Leading Innovation & Collaboration

JCTS COVID-19 Special Edition Survey

Hub PIs and Administrators,

Please send this document to those you believe are most knowledgeable at answering how your hub has responded to the COVID-19 pandemic in the following categories:

- Section 1: N3C and Informatics
- Section 2: Biorepositories
- Section 3: Virtual Visits
- Section 4: IRB
- Section 5: Protecting Research Personnel
- Section 6: Prioritizing COVID-19 Studies
- Section 7: COVID-19 Adaptations to Pharmacy Procedures
- Section 8: Prioritizing non-COVID-19 Studies

When you send this document please specify which of these sections they are responsible for. Also please request that the Word document is returned to you prior to the REDCap deadline to ensure that you have ample time to input the data.

If in addition to the completed REDCap online survey, your hub would like to send multiple responses to some questions, please complete the REDCap survey with the most detailed response and send **ONE** word document with the multiple responses (please indicate in the section header if there is more than one response) to survey@ctsa-clic.org, and we will forward those responses to the appropriate writing group.

Thank you for your help in completing the REDCap survey with the questions from the writing groups preparing the manuscripts for the CTSA JCTS COVID-19 Special Edition. The purpose of this survey is to solicit information on the creative innovations and adaptations made by the hubs in response to the COVID-19 pandemic.

This WORD DOCUMENT is to be used internally within each hub to solicit responses from several hub colleagues in their area of knowledge/expertise in order for you to complete the online REDCap survey.

Key

Square Brackets [ ] = Check all that apply

Parenthesis ( )= Choose only one response

Directions for conditional questions

The University of Rochester Center for Leading Innovation and Collaboration (CLIC) is the coordinating center for the Clinical and Translational Science Awards (CTSA) Program, funded by the National Center for Advancing Translational Sciences (NCATS) at the National Institutes of Health (NIH), Grant U24TR002260.

Contents

Section1: N3C and Informatics 3
Section 2: Biorepositories 7
Section 3: Virtual Visits 11
Section 4: IRB 13
Section 5: Protecting Research Personnel 15
Section 6: Prioritizing COVID-19 Studies 16
Section 7: COVID-19 Adaptations to Pharmacy Procedures 20
Section 8: Prioritizing Non-COVID-19 Studies 24

The University of Rochester Center for Leading Innovation and Collaboration (CLIC) is the coordinating center for the Clinical and Translational Science Awards (CTSA) Program, funded by the National Center for Advancing Translational Sciences (NCATS) at the National Institutes of Health (NIH), Grant U24TR002260.

Section1: N3C and Informatics

1. Does your organization have a dedicated data warehouse for research?
   a. ( ) Yes
   b. ( ) No

If you chose “a” for Question 1: Please answer 1.1, 1.2, & 1.3

1.1. What is the Common Data Model (CDM)? (Check all that apply)
   a. [ ] i2b2/ACT
   b. [ ] PCORNet
   c. [ ] TriNetX
   d. [ ] OMOP
   e. [ ] Other

1.1a. Please specify "Other" Common Data Model.

1.2. What was the refresh frequency pre-COVID-19?
   a. ( ) Daily
   b. ( ) Weekly
   c. ( ) Monthly
   d. ( ) Other

1.2a. Please specify "Other" refresh frequency pre-COVID-19.

1.3. How did this frequency change during the pandemic?
   a. ( ) More Frequent
   b. ( ) Less Frequent
   c. ( ) Stayed the Same

1.3a. Please describe the frequency change indicated above.

If you chose “b” for Question 1: Please answer 1.4

1.4. Please describe the resources for querying and analyzing the data.

The University of Rochester Center for Leading Innovation and Collaboration (CLIC) is the coordinating center for the Clinical and Translational Science Awards (CTSA) Program, funded by the National Center for Advancing Translational Sciences (NCATS) at the National Institutes of Health (NIH), Grant U24TR002260.

2. Are your researchers able to do rapid cohort identification of COVID-19 patients?
    a. ( ) Yes
    b. ( ) No

3. Has your organization developed a COVID-19 Data Mart/Registry and/or dashboard?
    a. ( ) Yes
    b. ( ) No

If you chose "a" for Question 3: Please answer 3.1

3.1 Please select what the COVID-19 Data Mart/Registry and/or dashboard is used for. (Check all that apply)
- a. [ ] Predictive Model Development
- b. [ ] Clinical Trials
- c. [ ] Operations
- d. [ ] Other

3.1a. Please specify "Other".

4. Have you seen an increase in the utilization of other informatics resources, such as REDCap, data warehouse, CTMS, management systems, etc. due to COVID-19?
    a. ( ) Yes
    b. ( ) Stayed the same
    c. ( ) No, we experienced a decrease

If you chose "a" or "c" for Question 4: Please answer 4.1

4.1 Please describe the resource and how it was used during COVID-19.

The University of Rochester Center for Leading Innovation and Collaboration (CLIC) is the coordinating center for the Clinical and Translational Science Awards (CTSA) Program, funded by the National Center for Advancing Translational Sciences (NCATS) at the National Institutes of Health (NIH), Grant U24TR002260.

5. Does your organization (Enterprise or CTSI) have a review committee(s) to manage the process for prioritization of access to EHR data?
   a. ( ) Yes
   b. ( ) No

If you chose "a" for Question 5: Please answer 5.1

5.1 Did this committee exist pre-pandemic?
   a. ( ) Yes
   b. ( ) No

If you chose "b" for Question 5.1: Please answer 5.1a & 5.1b

5.1a How did forming this committee help with COVID-19 research?

5.1b Please describe the committee members' areas of expertise and roles.

The University of Rochester Center for Leading Innovation and Collaboration (CLIC) is the coordinating center for the Clinical and Translational Science Awards (CTSA) Program, funded by the National Center for Advancing Translational Sciences (NCATS) at the National Institutes of Health (NIH), Grant U24TR002260.

6. In what capacity has your organization streamlined process for the following activities? (One choice per row)

One choice per column			COLUMN A	COLUMN B	COLUMN C	COLUMN D	COLUMN E
			**The process existed prior to the pandemic and adapted that process to support continuation of COVID-19 research during the pandemic**	**Implemented a new process as a result of the pandemic**	**Implemented a new process as a result of the pandemic AND will continue to use this process once research operations resume to normal**	**Streamlined a new process, but was not effective**	**No process exists at your institution**
ROW	A	**Data Request**	( )	( )	( )	( )	( )
ROW	B	**Review/Approval**	( )	( )	( )	( )	( )
ROW	C	**Delivery**	( )	( )	( )	( )	( )

If you chose Column "B" or "C" for any row in Question 6: Please answer 6.1

6.1. How did creating this process help with COVID-19 research?

The University of Rochester Center for Leading Innovation and Collaboration (CLIC) is the coordinating center for the Clinical and Translational Science Awards (CTSA) Program, funded by the National Center for Advancing Translational Sciences (NCATS) at the National Institutes of Health (NIH), Grant U24TR002260.

If you chose Column “A”, “B”, or “C” for any row in Question 6: Please answer 6.2

6.2 Please
describe the existing/newly implemented process and its impact on the COVID-19 research.

7. Describe any innovations that have emerged at your site due to COVID-19 in regards to informatics.

Section 2: Biorepositories

1. Which of the following best describes your biorepository model (Pre-COVID-19 and COVID-19 Specific) (One selection allowed per column)

One choice per column			COLUMN	
			A	B
			Pre-COVID-19	COVID-19 Specific
ROW	A	Individual, investigator-initiated biospecimen collection	( )	( )
	B	Enterprise approach with standard SOPs for biospecimen collection	( )	( )
	C	Hybrid Approach	( )	( )
	D	Other	( )	( )

1.1. Please describe “Other” biorepository model.

The University of Rochester Center for Leading Innovation and Collaboration (CLIC) is the coordinating center for the Clinical and Translational Science Awards (CTSA) Program, funded by the National Center for Advancing Translational Sciences (NCATS) at the National Institutes of Health (NIH), Grant U24TR002260.

2. Which of the following best describes your institution's biospecimen acquisition and banking consenting procedures? (Pre-COVID-19 and COVID-19 Specific) (check all that apply)

Check all that apply			COLUMN A Pre-COVID-19	COLUMN B COVID-19 Specific
ROW	A	Individual, investigator-initiated protocols and consents	[ ]	[ ]
	B	Enterprise approach for some but not all types of specimens (e.g., remnant surgical tissue)	[ ]	[ ]
	C	Enterprise approach with standard SOPs for all biospecimens	[ ]	[ ]
	D	Hybrid Approach	[ ]	[ ]
	E	Other	[ ]	[ ]

2.1. Please describe "Other" biospecimen acquisition and banking consenting procedures.

3. Which of the following describe changes made to your biospecimen acquisition and banking consenting procedures due to COVID-19? (Check all that apply)
   a. [ ] Continued with our pre-COVID-19 consenting procedures
   b. [ ] Developed a coordinated consent for all types of COVID-19 specimens proactively prepared the consenting process for non-English speakers
   c. [ ] Maintained a separation between specimens, clinical data, and permission for longitudinal re-contact
   d. [ ] Developed a coordinated consenting process for specimen acquisition, linkage to detailed clinical data, and longitudinal follow-up
   e. [ ] Other

3.1 Please specify "Other" changes made to your biospecimen acquisition and banking consenting procedures due to COVID-19.

4. Did your institution develop special governance to help manage acquisition and allocation of COVID-19 related biospecimens? (Check all that apply)
   a. [ ] Continued with our individual, investigator-initiated approach

The University of Rochester Center for Leading Innovation and Collaboration (CLIC) is the coordinating center for the Clinical and Translational Science Awards (CTSA) Program, funded by the National Center for Advancing Translational Sciences (NCATS) at the National Institutes of Health (NIH), Grant U24TR002260.

b. [ ] Created a governance group, which has guided the acquisition of investigator-requested samples and helped to prioritize sample allocation
c. [ ] In addition to governance, we created a scientific advisory group to anticipate specimen needs and obtain them proactively
d. [ ] Other
    4.1 Please specify "Other" developed governance to help manage acquisition and allocation of COVID-19 related biospecimens.

5. Which of the following group interests were prioritized when developing a governance and prioritization process to help balance internal vs. external requests for COVID-19 biospecimens? (Check all that apply)
    a. [ ] Academic colleagues
    b. [ ] Academic consortia
    c. [ ] Industry collaborators
    d. [ ] Other
        5.1. Please specify "Other" group interests that were prioritized when developing a governance and prioritization process.

6. How did your institution support expanded demands for your biorepository activities to accommodate COVID-19 related research? (Check all that apply)
    a. [ ] Individual investigator grants and charges
    b. [ ] Center grants and mechanisms, like the CTSA
    c. [ ] Funds from the School of Medicine, Health System, other schools
    d. [ ] Philanthropy
    e. [ ] Other
        6.1. Please specify "Other" institutional support for the expanded demands of biorepository activities.

7. What were the major obstacles that your institution encountered in enabling biorepository activities to support COVID-19 related research? (Check all that apply)
    a. [ ] IRB delays in approvals
    b. [ ] Resistance from investigators wanting their own IRB protocol
    c. [ ] Barriers in connecting biospecimens with detailed clinical data
    d. [ ] Other

The University of Rochester Center for Leading Innovation and Collaboration (CLIC) is the coordinating center for the Clinical and Translational Science Awards (CTSA) Program, funded by the National Center for Advancing Translational Sciences (NCATS) at the National Institutes of Health (NIH), Grant U24TR002260.

7.1 Please specify "Other" obstacles that your institution encountered in enabling biorepository activities to support COVID-19 related research.

8. What do anticipate will be the long term impact of COVID-19 on biorepositories at your institution?
   (Please consider the following)
   - Do you anticipate that more resources will be allocated to biobanking than before?
   - Will there be expanded use of e- or video-consent?
   - Will more BSL2+ lab space be made available to biorepositories?
   - Will there be changes in governance process?
   - Do you expect any negative impact?
   - Other?

9. Please provide any additional comments:
   (Please consider the following)
   - What recruitment strategies were more effective in the COVID-19 context?
   - What recruitment strategies were less effective in the COVID-19 context?
   - What consent strategies were more effective in the COVID-19 context?
   - What consent strategies were less effective in the COVID-19 context?
   - Did you encounter patient-specific considerations (e.g., special populations)?

Section 3: Virtual Visits

1. Were investigators at your institution able to utilize virtual approaches to support clinical research activities during the pandemic?
   a. ( ) Yes
   b. ( ) No

If you chose “a” in Question 1: Please answer 1.1

1.1. Describe your site's biggest challenges in moving to a virtual platform. (limit to 2)

If you chose “b” in Question 1: Please answer 1.2

1.2. What barriers hindered investigators at your institution from utilizing virtual approaches to support clinical research activities during the pandemic? (Check all that apply)

a. [ ] Funding Constraints
b. [ ] Institutional Policies/Procedures
c. [ ] Access to Technology
d. [ ] Regulatory/legal
e. [ ] Lack of provider interest or ethical concerns
f. [ ] Culturally related concerns per data integrity and fear of technical failings
g. [ ] Technical: Participant access to digital technology
h. [ ] Other

1.2a. Please specify "Other" barriers.

The University of Rochester Center for Leading Innovation and Collaboration (CLIC) is the coordinating center for the Clinical and Translational Science Awards (CTSA) Program, funded by the National Center for Advancing Translational Sciences (NCATS) at the National Institutes of Health (NIH), Grant U24TR002260.

2. Tell us about your institution's experience implementing a virtual process for conducting clinical study activities during the pandemic. (One choice per row)

One choice per row			COLUMN				
			A	B	C	D	E
			Institution had a virtual process prior to the pandemic and adapted that process during the pandemic	Implemented a new virtual process as a result of the pandemic	Implemented a new virtual process as a result of the pandemic AND will continue such process once normal research operations resume	Implemented, but was not effective	No virtual process exists at the institution
ROW	A	Participant Recruitment	( )	( )	( )	( )	( )
	B	Informed Consent	( )	( )	( )	( )	( )
	C	Study Visits	( )	( )	( )	( )	( )
	D	Investigational Product Management	( )	( )	( )	( )	( )
	E	Laboratory Sample Collection	( )	( )	( )	( )	( )
	F	Physiological Measures	( )	( )	( )	( )	( )
	G	Study Management	( )	( )	( )	( )	( )
	H	Study Monitoring/Audits	( )	( )	( )	( )	( )
	I	Delivery of Interventions	( )	( )	( )	( )	( )
	J	Other	( )	( )	( )	( )	( )

2.1a. Please specify "Other" virtual clinical study activities your institution experienced during the pandemic and its challenges if applicable.

If you chose Column “B” or “C” for any row (including “Other”) in Question 2: Please answer 2.2

2.2. Describe your site's leading innovation(s) in navigating to a virtual platform. (limit to 2)

The University of Rochester Center for Leading Innovation and Collaboration (CLIC) is the coordinating center for the Clinical and Translational Science Awards (CTSA) Program, funded by the National Center for Advancing Translational Sciences (NCATS) at the National Institutes of Health (NIH), Grant U24TR002260.

If you chose Column "D" any row (including "Other") in Question 2: Please answer 2.3

2.3. Please explain why the process(es) chosen above was not effective.

3. What digital technologies and platforms did you leverage? (limit to 2)

Section 4: IRB

1. Which statement best describes the process for IRB review of COVID-19 protocols at your institution?
   a. ( ) Created a separate IRB review committee for COVID-19 protocols
   b. ( ) Assigned all COVID-19 protocols to one existing IRB review committee
   c. ( ) COVID-19 protocols were reviewed by multiple IRB committees in the same way as other protocols pre-COVID
   d. ( ) Other
      1.1 Please specify "Other" IRB review of COVID-19 protocols.

2. Did your IRB review committee develop a new review process for COVID-19 related protocols?
   a. ( ) Yes, a new review process was implemented for COVID-19 related protocols
   b. ( ) Yes, a new review process was implemented AND is expected to be expanded to review of all protocols (even non-COVID studies)
   c. ( ) Yes, a new review process was implemented for COVID-19 related protocols but was not effective
   d. ( ) No new process was developed for COVID-19 related protocols

The University of Rochester Center for Leading Innovation and Collaboration (CLIC) is the coordinating center for the Clinical and Translational Science Awards (CTSA) Program, funded by the National Center for Advancing Translational Sciences (NCATS) at the National Institutes of Health (NIH), Grant U24TR002260.

If you chose option "b" in Question 2: Please answer 2.1

2.1. Can the expanded process to the review of all protocols (even non-COVID-19) be expanded without a substantial increase in resources?

a. ( ) Definitely yes
b. ( ) Possibly yes
c. ( ) No opinion
d. ( ) Possibly no
e. ( ) Definitely no

If you chose options "a", "b", or "c" for Question 2: Please answer 2.2 & 2.3

2.2. What is the current rate of the review process of COVID-19 related IRB protocols, compared to the rate of the review process of similar protocols before the COVID-19 pandemic?

a. ( ) Much faster
b. ( ) Somewhat faster
c. ( ) Same
d. ( ) Somewhat slower
e. ( ) Much slower

2.3. What is the quality of review of COVID related protocols compared to the quality of similar protocols before the COVID-19 pandemic?

a. ( ) Much better quality
b. ( ) Slightly better quality
c. ( ) Same
d. ( ) Slightly lower quality
e. ( ) Much lower quality

3. Roughly how many FTE did your IRB office utilize before the COVID-19 pandemic?

[ ]

4. Roughly how many additional FTE did your IRB office utilize to handle the COVID review demand during the pandemic?

[ ]

If you would like to provide further context on the IRB review process please contact Daniel Ford, MD, MPH dford@jhmi.edu

The University of Rochester Center for Leading Innovation and Collaboration (CLIC) is the coordinating center for the Clinical and Translational Science Awards (CTSA) Program, funded by the National Center for Advancing Translational Sciences (NCATS) at the National Institutes of Health (NIH), Grant U24TR002260.

Section 5: Protecting Research Personnel

1. How were COVID-19 related research governing policies disseminated at your institution? (Check all that apply)
   a. [ ] Workshops
   b. [ ] Emails
   c. [ ] Town halls
   d. [ ] Other
      1.1. Please specify "Other" research governing policies dissemination method.

2. Was your CTSA leadership involved in creating institutional COVID related governing policies?
   a. ( ) Yes
   b. ( ) No

3. Please share a link of your institution's policies regarding the protection of lab-related research staff.

4. Please share a link of your institution's policies regarding the protection of clinical research staff.

5. Please share a link of your institution's policies regarding the protection of students.

6. Please share a link of your institution's policies regarding the protection of postdoctoral fellows.

7. Are there lessons regarding protecting research personnel that you would like to share?

The University of Rochester Center for Leading Innovation and Collaboration (CLIC) is the coordinating center for the Clinical and Translational Science Awards (CTSA) Program, funded by the National Center for Advancing Translational Sciences (NCATS) at the National Institutes of Health (NIH), Grant U24TR002260.

If you would like to provide further context on the policies regarding the protection of faculty/staff please contact Rachel Hess, MD, MS Rachel.Hess@hsc.utah.edu

Section 6: Prioritizing COVID-19 Studies

1. Which of the following was most influential to your leadership for the decision making for developing an action plan to adjust research infrastructures to best adapt to pandemic related challenges?
   a. ( ) No action plan was developed
   b. ( ) Notification of the pandemic globally before the first U.S.
   c. ( ) Notification of the first COVID-19 Case in the U.S. case
   d. ( ) The peak of the pandemic in your area (use CDC definition of "peak")
   e. ( ) Other
      1.1. Please specify "Other".

2. Was the CTSA involved in the decision-making process?
   a. ( ) Yes
   b. ( ) No

If you chose "a" in Question 2: Please answer 2.1

2.1. How was the CTSA involved in the decision-making process?

3. If your institution was awarded emergency funding for COVID-19 research, what was the source? (Check all that apply)
   a. [ ] N/A. The institution did not receive funding
   b. [ ] Local CTSI
   c. [ ] State
   d. [ ] Federal
   e. [ ] Industry
   f. [ ] Institutional
   g. [ ] Philanthropic
   h. [ ] Special Interest Groups
   i. [ ] Other ___________________
      3.1 Please describe the "Other" source of emergency funding your institution received for COVID-19 research.

The University of Rochester Center for Leading Innovation and Collaboration (CLIC) is the coordinating center for the Clinical and Translational Science Awards (CTSA) Program, funded by the National Center for Advancing Translational Sciences (NCATS) at the National Institutes of Health (NIH), Grant U24TR002260.

If you chose “b” through “i” in Question 3: Please answer 3.2

3.2. Please describe the source of emergency funding your institution received for COVID-19 research.

4. Did the institution create a COVID-19 dedicated area of research?
   a. ( ) Yes
   b. ( ) No

If you chose “a” in Question 4: Please answer 4.1

4.1. Please explain.

5. Did the institution create COVID-19 laboratory services for rapid diagnosis for research and patient care?
   a. ( ) Yes
   b. ( ) No

If you chose “a” in Question 5: Please answer 5.1

5.1. What did the institution create to improve the diagnostics?

6. Did the institution enforce any intellectual property policies for COVID-19 specific research?
   a. ( ) N/A. There were no relevant intellectual property issues in relation to COVID -19 research
   b. ( ) No
   c. ( ) Yes

If you chose “c” in Question 6: Please answer 6.1

6.1 Did the enforcement of intellectual property policies for COVID-19 specific research cause disruptions in research?
   a. ( ) Yes
   b. ( ) No

The University of Rochester Center for Leading Innovation and Collaboration (CLIC) is the coordinating center for the Clinical and Translational Science Awards (CTSA) Program, funded by the National Center for Advancing Translational Sciences (NCATS) at the National Institutes of Health (NIH), Grant U24TR002260.

If you chose “a ” in Question 6.1: Please answer 6.1a

6.1a. Please explain how these intellectual property policies disrupts research at your institution.

7. Did the institution enforce any commercialization policies for COVID-19 specific research?
   a. ( ) N/A. There were no relevant commercialization policy issues in relation to COVID -19 research
   b. ( ) No
   c. ( ) Yes

If you chose “c” in Question 7: Please answer 7.1

7.1. Did the enforcement of commercialization policies for COVID-19 specific research cause disruptions in research?
   a. ( ) Yes
   b. ( ) No

If you chose “a ” in Question 7.1: Please answer 7.1a

7.1a. Please explain how these commercialization policies disrupts research at your institution.

8. Which of the following changes did human resources (HR) implement during the pandemic? (Check all that apply)
   a. [ ] None
   b. [ ] Furloughs
   c. [ ] Hiring freeze
   d. [ ] Restricted facility access/ hours
   e. [ ] Limited facility capacity / Personnel rotation
   f. [ ] Salary adjustments
   g. [ ] Staff hired for COVID-19 research
   h. [ ] Overtime pay

The University of Rochester Center for Leading Innovation and Collaboration (CLIC) is the coordinating center for the Clinical and Translational Science Awards (CTSA) Program, funded by the National Center for Advancing Translational Sciences (NCATS) at the National Institutes of Health (NIH), Grant U24TR002260.

i. [ ] Other

8.1. Please specify "Other".

If any of the options are chosen except for “a” in Question 8: Please answer 8.2

8.2. How did HR change its process to match the increased demand of labor needed for COVID-19 studies?

9. Did the institution establish any form of a feasibility committee to review all COVID-19 related studies in order to facilitate best practices?
   a. ( ) N/A. There were no COVID related studies conducted at our institution.
   b. ( ) Yes
   c. ( ) No
   d. ( ) Other

If you chose “b” or “d” in Question 9: Please answer 9.1

9.1. Please describe the feasibility committee that was established.

10. Are there any procedures or practices that you have implemented that worked?
    a. ( ) Yes, the institution implemented a new process as a result of the pandemic
    b. ( ) Yes, the institution implemented a new process **AND** is expected to continue with such procedures once research operations resume to normal
    c. ( ) No, the institution implemented a new process, but was not effective
    d. ( ) N/A, the institution did not implement a new process

If any of the options are chosen except “d” in Question 10: Please Answer 10.1

10.1 Please elaborate on your answer from the previous question.

The University of Rochester Center for Leading Innovation and Collaboration (CLIC) is the coordinating center for the Clinical and Translational Science Awards (CTSA) Program, funded by the National Center for Advancing Translational Sciences (NCATS) at the National Institutes of Health (NIH), Grant U24TR002260.

Section 7: COVID-19 Adaptations to Pharmacy Procedures

1. Did your pharmacy modify processes during the pandemic in the following areas? (Check all that apply)
   a. [ ] Research pharmacy services
   b. [ ] Pharmacy-based vaccination practice
   c. [ ] Pharmacy-based diagnostics and testing

2. What types of modifications were made to your research pharmacy services? Check all that apply.

Check all that apply			COLUMN		
			A	B	C
			COVID-19 related activities	Non-COVID-19 related activities	N/A
ROW	A	Leverage expertise to assist in rapid project startup development	[ ]	[ ]	[ ]
	B	Supply and dispense commercial drug products for use in drug repurposing research protocols	[ ]	[ ]	[ ]
	C	Supply or formulate Active Pharmaceutical Ingredient(s) for use in research protocols	[ ]	[ ]	[ ]
	D	Create product development teams	[ ]	[ ]	[ ]
	E	Other	[ ]	[ ]	[ ]

2.1. Please specify "Other" modification made to your research pharmacy services.

The University of Rochester Center for Leading Innovation and Collaboration (CLIC) is the coordinating center for the Clinical and Translational Science Awards (CTSA) Program, funded by the National Center for Advancing Translational Sciences (NCATS) at the National Institutes of Health (NIH), Grant U24TR002260.

3. What types of modifications were made to your Pharmacy-based vaccination practice? Check all that apply.

Check all that apply			COLUMN		
			A	B	C
			COVID-19 related activities	Non-COVID-19 related activities	N/A
ROW	A	Expand pharmacy-based vaccination practice in response to the Federal Public Readiness and Emergency Preparedness Act	[ ]	[ ]	[ ]
	B	Other	[ ]	[ ]	[ ]

3.1. Please specify "Other" Pharmacy-based vaccination practice modifications

4. What types of modifications were made to your Pharmacy-based diagnostics and testing practices? Check all that apply.

Check all that apply			COLUMN		
			A	B	C
			COVID-19 related activities	Non-COVID-19 related activities	N/A
ROW	A	Expand diagnostic testing	[ ]	[ ]	[ ]
	B	Other	[ ]	[ ]	[ ]

The University of Rochester Center for Leading Innovation and Collaboration (CLIC) is the coordinating center for the Clinical and Translational Science Awards (CTSA) Program, funded by the National Center for Advancing Translational Sciences (NCATS) at the National Institutes of Health (NIH), Grant U24TR002260.

4.1. Please specify "Other" modifications that were made to your pharmacy-based diagnostic and testing practices.

5. Did you experience any shortages in the following areas? (Check all that apply)
   a. [ ] Drug Shortages
   b. [ ] PPE Shortage

6. What types of modifications were made at a pharmacy level to adapt to drug shortages? Check all that apply.

Check all that apply			COLUMN		
			A	B	C
			COVID-19 related activities	Non-COVID-19 related activities	N/A
ROW	A	New vendors/distributers/suppliers	[ ]	[ ]	[ ]
	B	Preemptive drug stockpiling	[ ]	[ ]	[ ]
	C	Develop protocol for drug prioritization	[ ]	[ ]	[ ]
	D	In-house drug compounding	[ ]	[ ]	[ ]
	E	In-house drug supply centralization	[ ]	[ ]	[ ]
	F	Limit distribution of prescription drugs for off label use based on social media claims	[ ]	[ ]	[ ]
	G	Other	[ ]	[ ]	[ ]

6.1. Please describe "Other" modifications made at a pharmacy level to adapt to drug shortages.

The University of Rochester Center for Leading Innovation and Collaboration (CLIC) is the coordinating center for the Clinical and Translational Science Awards (CTSA) Program, funded by the National Center for Advancing Translational Sciences (NCATS) at the National Institutes of Health (NIH), Grant U24TR002260.

7. What types of modifications were made at a pharmacy level to adapt to PPE shortages? Check all that apply.

Check all that apply			COLUMN		
			A	B	C
			COVID-19 related activities	Non-COVID-19 related activities	N/A
ROW	A	New vendors/distributers/suppliers for PPE	[ ]	[ ]	[ ]
	B	Preemptive PPE stockpiling	[ ]	[ ]	[ ]
	C	Develop protocol for PPE prioritization	[ ]	[ ]	[ ]
	D	In-house PPE creation/adaption	[ ]	[ ]	[ ]
	E	In-house PPE supply centralization	[ ]	[ ]	[ ]
	F	Limit PPE distribution	[ ]	[ ]	[ ]
	G	Other	[ ]	[ ]	[ ]

7.1. Please describe "Other" modification were made at a pharmacy level to adapt to PPE shortages.

8. Please provide any additional comments you may have.

If you would like to provide further context regarding your responses directly, please contact Robert B. MacArthur, PharmD, MS, BCSCP rmacarthur@rockefeller.edu

The University of Rochester Center for Leading Innovation and Collaboration (CLIC) is the coordinating center for the Clinical and Translational Science Awards (CTSA) Program, funded by the National Center for Advancing Translational Sciences (NCATS) at the National Institutes of Health (NIH), Grant U24TR002260.

Section 8: Prioritizing Non-COVID-19 Studies

1. Between January 1, 2020 and September 30, 2020 did your institution at any point mandate a reduction in either of the following research activities? Check all that apply.
   a. [ ] Laboratory Research
   b. [ ] Human Subjects Research
   c. [ ] NA

Laboratory Research Adaptations Due to COVID-19

2. Who made the decisions regarding cessation of laboratory research activity? Check all that apply
   a. [ ] President or Provost Office
   b. [ ] College/School decision
   c. [ ] Department decision
   d. [ ] Division decision
   e. [ ] Other

   2.1. Please describe other entities involved in decisions regarding cessation of laboratory research activity.

3. What was the involvement of the CTSA in the decisions regarding the cessation of laboratory research?
   a. ( ) No involvement
   b. ( ) No part in decision making, key role in implementation
   c. ( ) Part of the decision making, no role in implementation
   d. ( ) Part of decision making, key role in implementation
   e. ( ) Other

   3.1. Please describe "Other" involvement of the CTSA regarding the cessation of laboratory research.

The University of Rochester Center for Leading Innovation and Collaboration (CLIC) is the coordinating center for the Clinical and Translational Science Awards (CTSA) Program, funded by the National Center for Advancing Translational Sciences (NCATS) at the National Institutes of Health (NIH), Grant U24TR002260.

4. Please describe your institution's policies regarding a reduction in laboratory research activity due to COVID-19 in the following time frames. If your institution updated policies in the middle of a time frame, check both options that apply.

Check all that apply			COLUMN		
			A	B	C
			Complete cessation of all research activity	Partial cessation of research activity	No cessation of research activity (continue normal activity)
ROW	A	Prior to January 2020	[ ]	[ ]	[ ]
	B	January-March 2020	[ ]	[ ]	[ ]
	C	April-June 2020	[ ]	[ ]	[ ]
	D	July-September 2020	[ ]	[ ]	[ ]

If you chose "Column B" for any of the rows: Continue to Question 4.1
If you only chose "Column A" or "Column C" in Question 4: Skip to Question 5

4.1. What laboratory research activities were allowed to continue? Check all that apply

Check all that apply			COLUMN				
			A	B	C	D	E
			Prior to January 2020	January-March 2020	April-June 2020	July-September 2020	NA
ROW	A	Only lab maintenance (e.g. keeping cell lines alive) but no new experiments	[ ]	[ ]	[ ]	[ ]	[ ]
	B	Limited to spaces that already have sufficient space to allow physical distancing	[ ]	[ ]	[ ]	[ ]	[ ]
	C	Only with reduced personnel to allow for physical distancing	[ ]	[ ]	[ ]	[ ]	[ ]
	D	Depended on funding source (e.g. federal)	[ ]	[ ]	[ ]	[ ]	[ ]
	E	Other	[ ]	[ ]	[ ]	[ ]	[ ]

4.1a. Please specify "other" laboratory research activities that were allowed to continue.

The University of Rochester Center for Leading Innovation and Collaboration (CLIC) is the coordinating center for the Clinical and Translational Science Awards (CTSA) Program, funded by the National Center for Advancing Translational Sciences (NCATS) at the National Institutes of Health (NIH), Grant U24TR002260.

4.2. Please provide any additional or clarifying comments you have about partial cessation of laboratory research activities, if necessary.

5. Have you since allowed resumption of laboratory research?
   a. ( ) Yes, back to previous activity unrestricted
   b. ( ) Yes, all types of studies have resumed that have met resumption criteria
   c. ( ) Yes, limited types of studies have resumed that have met resumption criteria
   d. ( ) No, restrictions have not been lifted

For any response choice in Question 5: Please answer 5.1, 5.2., & 5.3

5.1. Was a specific approval process required in order to resume laboratory research activities?
   a. ( ) No requirements
   b. ( ) Approval request had to be submitted
   c. ( ) Other

5.1a. Please describe "Other" approval process for resuming laboratory research.

5.2. Who made the decisions regarding resumption of laboratory research activity? Check all that apply
   a. [ ] President or Provost Office
   b. [ ] College/School decision
   c. [ ] Department decision
   d. [ ] Division decision
   e. [ ] Other

5.2a. Please describe "Other" entities involved in decisions regarding resumption of laboratory research activity.

5.3 What was the involvement of the CTSA in the decisions regarding the resumption of laboratory research?

a. ( ) No involvement
b. ( ) No part in decision making, key role in implementation
c. ( ) Part of the decision making, no role in implementation
d. ( ) Part of decision making, key role in implementation
e. ( ) Other

5.3a. Please describe "Other" involvement of the CTSA regarding the resumption of laboratory research.

If you chose “b” or “c: for Question 5: Please answer 5.4 & 5.5
If you chose “a” or “d” for Question 5

- **AND “b” for Question 1: Continue to Question 6**
- **Otherwise: Skip to Question 10**

5.4 What criteria must be met for laboratory research activities to resume? Check all that apply

a. [ ] Limited personnel (e.g. 50% of baseline)
b. [ ] Sufficient personal protective equipment
c. [ ] Negative COVID testing for personnel
d. [ ] Daily symptom reporting
e. [ ] Other

5.4a. Please describe "Other" criteria for laboratory research resumption.

The University of Rochester Center for Leading Innovation and Collaboration (CLIC) is the coordinating center for the Clinical and Translational Science Awards (CTSA) Program, funded by the National Center for Advancing Translational Sciences (NCATS) at the National Institutes of Health (NIH), Grant U24TR002260.

5.5 Please describe what limited types of laboratory research have been allowed to resume.

Human Subjects Research Adaptations Due to COVID-19

6. Who made the decisions regarding cessation of human subjects research activity?
   a. [ ] President or Provost Office
   b. [ ] College/School decision
   c. [ ] Department decision
   d. [ ] Division decision
   e. [ ] Other
      6.1. Please describe “Other” entities involved in decision regarding cessation of human subjects research activity.

7. What was the involvement of the CTSA in the decisions regarding the cessation of human subjects research?
   a. ( ) No involvement
   b. ( ) No part in decision making, key role in implementation
   c. ( ) Part of the decision making, no role in implementation
   d. ( ) Part of decision making, key role in implementation
   e. ( ) Other
      7.1. Please describe "other" involvement of the CTSA regarding the cessation of human subjects research.

The University of Rochester Center for Leading Innovation and Collaboration (CLIC) is the coordinating center for the Clinical and Translational Science Awards (CTSA) Program, funded by the National Center for Advancing Translational Sciences (NCATS) at the National Institutes of Health (NIH), Grant U24TR002260.

8. Please describe your institution's policies regarding a reduction in human subjects research activity due to COVID-19 in the following time frames. If your institution updated policies in the middle of a time frame, check both options that apply.

Check all that apply			COLUMN A	COLUMN B	COLUMN C
			Complete cessation of all research activity	Partial cessation of research activity	No cessation of research activity (continue normal activity)
ROW	A	Prior to January 2020	[ ]	[ ]	[ ]
ROW	B	January-March 2020	[ ]	[ ]	[ ]
ROW	C	April-June 2020	[ ]	[ ]	[ ]
ROW	D	July-September 2020	[ ]	[ ]	[ ]

If you chose "Column B" for any of the rows: Please answer 8.1
If you only chose "Column A" or "Column C" in Question 9: Skip to Question 9

8.1 What human subjects research activities were allowed to continue? Check all that apply

Check all that apply			COLUMN A	COLUMN B	COLUMN C	COLUMN D	COLUMN E
			Prior to January 2020	January-March 2020	April-June 2020	July-September 2020	NA
ROW	A	Only studies that could be completed without direct subject contact	[ ]	[ ]	[ ]	[ ]	[ ]
ROW	B	All types of studies with in-person activities	[ ]	[ ]	[ ]	[ ]	[ ]
ROW	C	Limited types of studies with in-person activities	[ ]	[ ]	[ ]	[ ]	[ ]
ROW	D	All types of studies with currently enrolled subjects	[ ]	[ ]	[ ]	[ ]	[ ]
ROW	E	Limited types of studies with currently enrolled subjects	[ ]	[ ]	[ ]	[ ]	[ ]
ROW	F	Subject recruitment for all types of studies	[ ]	[ ]	[ ]	[ ]	[ ]
ROW	G	Subject recruitment for limited types of studies	[ ]	[ ]	[ ]	[ ]	[ ]
ROW	H	Only studies that could be completed without direct subject contact	[ ]	[ ]	[ ]	[ ]	[ ]
ROW	I	Other	[ ]	[ ]	[ ]	[ ]	[ ]

8.1a Please specify "Other" human subjects research activities that were allowed to continue.

The University of Rochester Center for Leading Innovation and Collaboration (CLIC) is the coordinating center for the Clinical and Translational Science Awards (CTSA) Program, funded by the National Center for Advancing Translational Sciences (NCATS) at the National Institutes of Health (NIH), Grant U24TR002260.

If you chose Column A,B, C, or D for Rows A, C or E in Question 8.1: Please answer 8.1.1

8.1.1 What criteria were used to determine which types of human subject research activities were allowed to continue in a limited capacity?

a. [ ] Studies that were associated with standard of care (e.g. inpatient studies)
b. [ ] Studies using life-saving interventions only (e.g. cancer therapies)
c. [ ] Other

8.1.1a Please specify "Other" criteria that were used to determine which human subject research activities were allowed to continue in a limited capacity.

If you chose Column A,B, C, or D for Row G in Question 8.1: Please answer 8.1.2

8.1.2 What criteria were used to determine which human subject research studies were allowed to enroll subjects?

a. [ ] Studies that were associated with standard of care (e.g. inpatient studies)
b. [ ] Studies using life-saving interventions only (e.g. cancer therapies)
c. [ ] Other

8.1.2a Please specify "Other" criteria that were used to determine which human subject research studies were allowed to continue enrolling subjects.

8.2 Please provide any additional or clarifying comments you have about partial cessation of human subjects research activities, if necessary.

The University of Rochester Center for Leading Innovation and Collaboration (CLIC) is the coordinating center for the Clinical and Translational Science Awards (CTSA) Program, funded by the National Center for Advancing Translational Sciences (NCATS) at the National Institutes of Health (NIH), Grant U24TR002260.

9. Have you since allowed resumption of human subjects research?
   a. ( ) Yes, back to previous activity unrestricted
   b. ( ) Yes, all types of studies have resumed that have met resumption criteria
   c. ( ) Yes, limited types of studies have resumed that have met resumption criteria
   d. ( ) No, restrictions have not been lifted

For any response choice in Question 9: Please answer 9.1, 9.2., & 9.3

9.1 Was specific approval process required in order to resume human subjects research activities?
   a. ( ) No requirements
   b. ( ) Approval request had to be submitted
   c. ( ) Other

9.1a. Please describe "Other" approval process for resuming human subjects research.

9.2 Who made the decisions regarding resumption of human subjects research activity?
   a. [ ] President or Provost Office
   b. [ ] College/School decision
   c. [ ] Department decision
   d. [ ] Division decision
   e. [ ] Other

9.2a. Please describe "Other" entities involved in decision regarding resumption of human subjects research activity.

9.3 What was the involvement of the CTSA in the decisions regarding the resumption of human subjects research?
   a. ( ) No involvement
   b. ( ) No part in decision making, key role in implementation
   c. ( ) Part of the decision making, no role in implementation
   d. ( ) Part of decision making, key role in implementation
   e. ( ) Other

The University of Rochester Center for Leading Innovation and Collaboration (CLIC) is the coordinating center for the Clinical and Translational Science Awards (CTSA) Program, funded by the National Center for Advancing Translational Sciences (NCATS) at the National Institutes of Health (NIH), Grant U24TR002260.

9.3a. Please describe "Other" involvement of the CTSA regarding the resumption of human subjects research.

If you chose “b” or “c: for Question 9: Please answer 9.4
If you chose “a” or “d” for Question 9: Skip to Question 10

9.4 What criteria must be met for human subjects research activities to resume? Check all that apply

a. [ ] Limited personnel (e.g. 50% of baseline)
b. [ ] Sufficient personal protective equipment
c. [ ] Negative COVID testing for personnel
d. [ ] Daily symptom reporting
e. [ ] Other

9.4a Please describe “Other” criteria for human subjects research resumption.

10. Are there any procedures or practices that you have implemented that will remain in place after the emergency is over?

11. What key steps would you advise again if there is another emergency?

The University of Rochester Center for Leading Innovation and Collaboration (CLIC) is the coordinating center for the Clinical and Translational Science Awards (CTSA) Program, funded by the National Center for Advancing Translational Sciences (NCATS) at the National Institutes of Health (NIH), Grant U24TR002260.

12. What would you have done differently to deal with this emergency?

The University of Rochester Center for Leading Innovation and Collaboration (CLIC) is the coordinating center for the Clinical and Translational Science Awards (CTSA) Program, funded by the National Center for Advancing Translational Sciences (NCATS) at the National Institutes of Health (NIH), Grant U24TR002260.
